# Students’ Acceptance of Technology-Mediated Teaching – How It Was Influenced During the COVID-19 Pandemic in 2020: A Study From Germany

**DOI:** 10.3389/fpsyg.2021.636086

**Published:** 2021-01-28

**Authors:** Gergana Vladova, André Ullrich, Benedict Bender, Norbert Gronau

**Affiliations:** ^1^Chair of Business Informatics, Processes and Systems, University of Potsdam, Potsdam, Germany; ^2^Research Group Education and Advanced Training in the Digital Society, Weizenbaum Institute for the Networked Society, Berlin, Germany

**Keywords:** COVID-19, digital learning, discipline differences, e-learning, TAM, technology acceptance, technology-mediated teaching, university teaching

## Abstract

In response to the impending spread of COVID-19, universities worldwide abruptly stopped face-to-face teaching and switched to technology-mediated teaching. As a result, the use of technology in the learning processes of students of different disciplines became essential and the only way to teach, communicate and collaborate for months. In this crisis context, we conducted a longitudinal study in four German universities, in which we collected a total of 875 responses from students of information systems and music and arts at four points in time during the spring–summer 2020 semester. Our study focused on (1) the students’ acceptance of technology-mediated learning, (2) any change in this acceptance during the semester and (3) the differences in acceptance between the two disciplines. We applied the Technology Acceptance Model and were able to validate it for the extreme situation of the COVID-19 pandemic. We extended the model with three new variables (time flexibility, learning flexibility and social isolation) that influenced the construct of perceived usefulness. Furthermore, we detected differences between the disciplines and over time. In this paper, we present and discuss our study’s results and derive short- and long-term implications for science and practice.

## Introduction

Digital technologies have provided support in diverse policy, business, and societal application areas in the COVID-19 outbreak, such as pandemic management (Radanliev et al., 2020b), corporate communications (Camilleri, 2020), analysis of research data (Radanliev et al., 2020a), and education (Crawford et al., 2020). COVID-19 started as a global infectious disease in the spring of 2020, but the necessary measures to control the virus went beyond treatment and were also directed against its spread. Thus, for months, all interpersonal relationships were characterized by social distancing, and the pandemic raised not only medical but also social, economic and technological issues, among others. Higher education was one domain that the pandemic affected radically (Nuere and de Miguel, 2020; Watermeyer et al., 2020). During the worldwide lockdown, higher educational institutions had to immediately switch their activities from the classroom and the campus to a virtual space, which was the only alternative to a complete incapacity to act (Crawford et al., 2020; Kamarianos et al., 2020; Karalis and Raikou, 2020; Owusu-Fordjour et al., 2020; Shah et al., 2020).

University students represent a generation of digital natives for whom this steady switch from the real to the virtual world should not pose any operational challenge (Carlson, 2005; Berk, 2009; Jones et al., 2010). However, research indicates that students show differences according to discipline, such as subject matter (Biglan, 1973; Neumann, 2001) or facets of digital literacy and competency (Nelson et al., 2011), which should be taken into consideration when developing digital learning environments and approaches. The issue of whether and how teaching and learning differs across disciplines has however long been neglected in academic discourse (Neumann, 2001). Furthermore, as in any field, the successful introduction of technology into existing processes – such as the phenomenon that occurred in the COVID-19 pandemic during the spring–summer 2020 semester (or the so-called COVID-19 semester) – can only be guaranteed if teachers and students show or develop appropriate attitudes, beliefs, behaviors and habits (Al-alak and Alnawas, 2011; Al-Harbi, 2011).

Starting from the circumstances of the pandemic – a rapid transition to fully technology-mediated teaching for students taking different subjects, with no alternative, accompanied by several months of social isolation – in this paper, we ask:

Do the acceptance toward completely technology-mediated teaching differ, depending on the discipline of study?

Did the students’ acceptance toward completely technology-mediated teaching change over time during the COVID-19 semester?

To address the research questions, we empirically examine the acceptance of technology-mediated teaching by students during the COVID-19 semester in the spring-summer of 2020. We follow the suggestion of Neumann (2001) that “the strong influence of disciplines on […] students’ learning” creates the need “disciplines to be subjected to greater systematic study, especially regarding their effect on the quality of teaching and learning in higher education,” and present, analyze and discuss the collected data from 875 responses gathered from students of two disciplines (information systems [IS] and music and arts [M&A]) at four points in time.

For our empirical investigation, we apply an extended version of the Technology Acceptance Model (TAM). Technology acceptance is a main topic in information systems (IS) research, and TAM is a widely used approach to investigating a subject’s attitude and adoption behavior, *inter alia* in university context (Venkatesh and Davis, 2000; Lee et al., 2005; Pituch and Lee, 2006; Al-Azawei et al., 2017). For the purpose of this study, the model allows us to investigate the acceptance of technology-mediated teaching, especially regarding certain aspects (usefulness, ease of use and enjoyment) that are relevant for students. Our goal is to understand not only whether students accept technology-mediated teaching but also what key aspects are decisive for the future design of technology-mediated teaching environments. For this reason, we apply the TAM, as well as look beyond the model at the research on the advantages and the disadvantages of technology-mediated teaching and the extended TAM, using three new variables to be able to analyze the construct of perceived usefulness for students during the COVID-19 season in more detail and depth.

This paper is organized as follows: In Section “Theoretical Framework,” we discuss the theoretical foundations of our investigation. The design and the procedure of the study, as well as the measures and data analysis are presented in Section “Materials and Methods.” The presentation of the results is the focus of Section “Results.” We discuss the results of the analysis in Section “Discussion of the Results” and provide implications for teaching practice and organization, educational technology, and research in Section “Implications for Teaching Practice, Educational Technology and Further Research.” We conclude this paper in Section “Conclusion” with a short summary, limitations of the study, and remarks on future studies.

## Theoretical Framework

### Technology Acceptance Model (TAM)

The TAM is one of the most widely investigated and applied models of technology acceptance. Perceived usefulness (PU) and perceived ease of use (PEOU) are the two decisive variables for a person’s attitude (ATT) toward a used technology, which in turn affects the actual system use. PU depicts a person’s subjective sensation that the application of a certain technology improves individual work performance, while PEOU measures a person’s perception of how much effort the usage of the new technology requires. Both variables are influenced by diverse external variables, such as job relevance, subjective norm or output quality (Venkatesh and Davis, 2000). Davis et al. (1989) adjusted the model by adding a person’s behavioral intention (BI) as mediator between ATT and actual system use.

Table 1 shows an overview of the research on the TAM in the e-learning context. For the e-learning context, Lee et al. (2005) added perceived enjoyment (PE) as an intrinsic motivator, in addition to PU and PEOU, to TAM constructs. Šumak et al. (2011) conducted a meta-analysis and found that the TAM was the most applied model in e-learning and that the size of the causal effects between individual TAM-related factors depends on *the type of user* and *the type of e-learning technology*.

**TABLE 1:** Previous research related to TAM in e-learning.

**Study**	**Constructs**	**Method**	**Key findings**
Šumak et al., 2011	perceived usefulness, perceived ease of use, attitude toward using, behavioral intention, usage, self-efficacy, satisfaction, social influence, compatibility, facilitating conditions, performance expectancy, confirmation, experience, system quality, anxiety, computer self-efficacy, management support, and flow	Meta-analysis	TAM is the most applied model in e-learning. The size of the causal effects between individual TAM-related factors depends on the type of user and the type of e-learning technology. PEOU and PU influence the user attitudes toward using an e-learning technology in equal measure for different user types and types of e-learning technology settings
Selim, 2003	perceived usefulness, perceived ease, course website ease of use, course website usefulness, course website usage	SEM of LISREL	usefulness and ease of use are good determinants of the acceptance course websites are an effective and efficient learning technology
Lee et al., 2005	perceived usefulness, perceived ease of use, perceived enjoyment, attitude, behavioral intention	SEM of LISREL VIII	perceived usefulness and perceived enjoyment had an impact on both students’ attitude toward and intention to use ILM. Perceived ease of use was found to be unrelated to attitude.
Liu et al., 2005	e-learning presentation types, perceived usefulness, perceived ease of use, attitude, intention	repeated-measures one-way ANOVA test with the independent variable	Dual identity of the online e-learning user as a system user and a learner was confirmed. Both the flow and the perceived usefulness of the e-learning system strongly predict intention to continue using e-learning
Pituch and Lee, 2006	system characteristics, learner characteristics, perceived usefulness, perceived ease of use, use of an e-learning system	SEM	E-learning presentation type and users’ intention to use e-learning were related to one another. Concentration and perceived usefulness were considered intermediate variables
Park, 2009	e-learning self-efficacy, subjective norm, system accessibility, perceived usefulness, perceived ease of use, attitude, and behavioral intention to use e-learning	SEM	TAM to be a good theoretical tool to understand users’ acceptance of e-learning. E-learning self-efficacy was the most important construct, followed by subjective norm in explicating the causal process in the model
Tarhini et al., 2013	social norms, quality of work life, perceived usefulness, perceived ease of use, attitude, behavioral intention, usage	SEM	Analysis results reveal that all the hypotheses are supported
Mohammadi, 2015	quality features, perceived ease of use, perceived usefulness on users’ intentions, satisfaction, usability toward use of e-learning	SEM, path analysis	‘Intention” and “user satisfaction” both had positive effects on actual use of e-learning. “System quality” and “information quality” were found to be the primary factors driving users’ intentions and satisfaction toward use of e-learning. “Perceived usefulness” mediated the relationship between ease of use and users’ intentions
Al-Azawei et al., 2017	e-learning self-efficacy, perceived satisfaction, learning styles, perceived usefulness, perceived ease of use, intention to use	PLS SEM	Highlights the integration of perceived satisfaction and technology acceptance in accordance with psychological traits and learner beliefs. Model achieved an acceptable fit and successfully integrated intention to use (ITU) and perceived satisfaction

**FIGURE 1:**
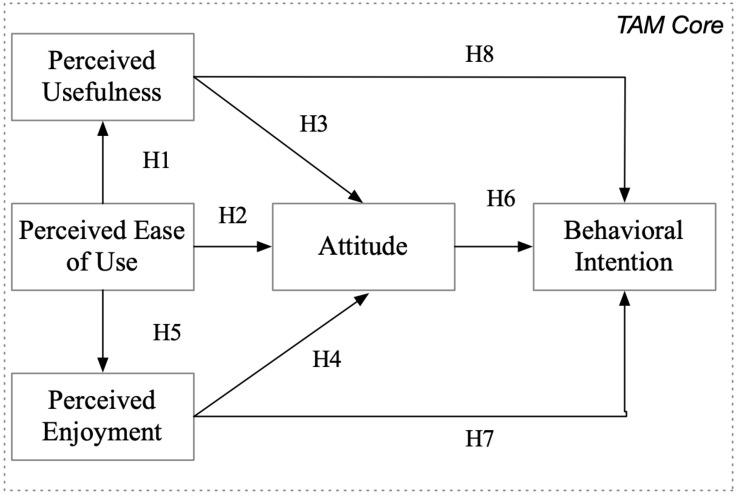
Research model (adapted from Lee et al., 2005).

For our study, we adapt the research model of Lee et al. (2005), as presented in Figure 1.

Consistent with the findings of prior studies (cf. Davis et al., 1989; Lee et al., 2005), we expect the relations among the constructs to exhibit significant strength (for the list of the hypotheses, cf. Attachment 1). However, in our discussion, we take into account that the TAM in e-learning is usually researched in cases where blended learning or e-learning is an additional part of face-to-face teaching, whereas in the COVID-19 semester, virtual teaching and learning was the only channel used to convey content. We examine the measurement model and the structural model and then compare the results over time and for the two student populations (IS and M&A).

#### Acceptance Over Time

Venkatesh and Davis (2000) tested an extended TAM (TAM2) in four longitudinal studies and introduced experience as a relevant influencing factor that is important for understanding the changes in PU over time, whereby experience in general reflects an opportunity to use a technology and is typically operationalized as the passage of time from an individual’s initial use of a technology. Based on the TAM, Venkatesh et al. (2003) developed UTAUT and tested it in a longitudinal field study. Venkatesh et al. (2012) introduced three new constructs to UTAUT, measured users’ experience and investigated its influence on the users’ acceptance and habits.

Davis and Wong (2007) applied TAM2 in an educational context and measured users’ experience in relation to the actual student usage (system use) of an e-learning system. They pointed out the complex underlying interactions during e-learning adoption processes and recommended a longitudinal design as appropriate for future studies. Pynoo et al. (2011) applied UTAUT in the educational context to investigate the acceptance of digital learning environments and found differences over time; they also pointed out that the usefulness of digital learning environments should be demonstrated to maximize its use.

In contrast to other studies, our study does not focus on a specific technology but on the experience with the technology-mediated teaching in the COVID-19 context. We expect and show within this context that students gain experience during the semester, which will lead to measurable changes in their acceptance.

#### Differences in Acceptance Between Student Groups

Hu et al. (1999) define a set of users’ characteristics as one factor that can be used to explain, predict and effectively manage technology acceptance.

Biglan (1973) points out the characteristics of academic matter, according to which the strongest differences between the “hard” (e.g., engineering sciences) and the “soft” sciences (e.g., social sciences, educational sciences, and humanities) can be identified. Vo et al. (2020) investigated the effects of blended learning on student learning performance and compared the output of students in hard and soft disciplines. According to their study, students in soft disciplines perform better than their peers in hard disciplines when courses are designed in the blended learning modality. Cameron (2017) identified differences in student engagement between ‘humanities’ (e.g., M&A) and ‘professional fields’ students (such as IS). Additionally, teaching experiences are more highly regarded by humanities students than those in hard sciences (Cashin and Downey, 1995). In the context of our study, this is expected to lead to differentiating results when lecturers had to quickly change toward virtual formats based on their diverse levels of experience with technologies. Pike et al. (2012) found that that students’ academic majors are significantly related to levels of engagement, which is influenced by their acceptance and learning outcomes. Students of enterprising disciplines are more engaged than artistic discipline students. Students of soft applied knowledge (e.g., M&A) need more intensive practical training than those from the disciplines of hard applied knowledge (e.g., IS) (Neumann et al., 2002). Here might be a major disadvantage for specific groups when virtual teaching is applied for learning.

According to the research on the learning characteristics and the learning styles of the Net generation (born after 1980) and the Z generation (millennials), university students at the time of the COVID-19 crisis are digital natives, who can be described as tending toward independence and autonomy in their learning styles, technology savvy, interested in communicating visually and in multimedia and able to move seamlessly between real and virtual worlds (Carlson, 2005; Berk, 2009; Jones et al., 2010). Despite this, it is also characteristic of this generation to view class as a social opportunity and to crave face-to face social interaction, whereby relationships, in-person conversation, interaction and collaboration are high priorities (Howe, 2000; Carlson, 2005; Ramaley and Zia, 2005). Zheng et al. (2017) investigated low- and high-performing students in an e-learning environment and identified a significant difference in the students’ perceived usefulness. Xu and Jaggars (2014) found that the typical student had more difficulty with succeeding in online courses than in face-to-face courses [compare also (Nelson et al., 2011); they also noted a variation across subject areas in terms of online course effectiveness].

To the best of our knowledge, to date, no research has put the TAM in the context of the specific characteristics of a study’s subjects. This is where our study can make a contribution, as we have examined two different subject groups: M&A students and IS students.

In summary, we expect differences in the students’ attitudes toward virtual learning, according to their academic subject.

### Benefits and Disadvantages of E-Learning

The benefits and the disadvantages of technology-mediated teaching and learning became a focal point for university research in the context of the COVID-19 crisis (Kamarianos et al., 2020; Karalis and Raikou, 2020; Owusu-Fordjour et al., 2020; Shah et al., 2020). However, this topic is not new but one of the central research focuses in the context of learning in digital learning environments. Davis and Wong (2007) define e-learning as a global phenomenon for organizations and educational institutions, aiming to enhance students’ learning experience and effectiveness in terms of the learning outcome. The benefits of e-learning have been discussed in recent research, but so far, there is no consensus on whether the outputs of e-learning are more effective than those of traditional learning formats (Derouin et al., 2005). The most frequently stated benefits are cost efficiency, flexibility (in terms of time and place), saving time to travel to the learning location, easy access to learning materials, as well as the usefulness of learning materials for a longer period (Welsh et al., 2003; Brown et al., 2006; Hameed et al., 2008; Jefferson and Arnold, 2009; Hill and Wouters, 2010; Al-Qahtani and Higgins, 2013; Becker et al., 2013), or the potential to offer personalized learning according to the learner’s specific needs (Berge and Giles, 2006).

On the negative side, technology-mediated learning lacks direct social interaction and a personal touch and has the potential to socially isolate the learner or at least to negatively influence social aspects of learning processes (Gimson and Bell, 2007; Hameed et al., 2008; Al-Qahtani and Higgins, 2013; Becker et al., 2013). Socially isolated learning can negatively influence the development of learners’ communication skills, as well as change communication conditions, including the lack of support and feedback using non-verbal cues or by observing the interactions of others, as well as the lack of social and cognitive presence and teacher’s involvement (Al-Qahtani and Higgins, 2013). Furthermore, learners are insecure about their learning in the absence of regular contact to the teachers (Al-Qahtani and Higgins, 2013). Technology-mediated teaching and learning requires self-motivation, time management and a focused approach and self-directed learning and organization skills of learners (Hameed et al., 2008; Jefferson and Arnold, 2009). According to Al-Qahtani and Higgins (2013), these requirements arise partly from the conditions of social isolation and lack of direct social interaction, which means that the learner must have a relatively strong motivation to mitigate this effect.

During the lockdown of the universities the expectation was that most of the young students will not have any difficulty in switching to online teaching, which is indeed confirmed by actual findings (e.g., Kamarianos et al., 2020). Shah et al. (2020) point out the numerous and immediately apparent benefits of transferring learning to the virtual world: free exchange of information, access to lectures and presentations at conferences that used to involve considerable travel costs, webinars and online discussions, reduction of time inefficiency associated with travel and increased commitment. Owusu-Fordjour et al. (2020) identify negative effects, e.g., learning at home can be ineffective because of many distractions, no adequate learning environment, or contact with the teacher. Less problems have been found in switching to online teaching, however, on the negative side, technical obstacles as well as lack of communication and cooperation, difficulties to concentrate, too many screen-time, lack of logistical infrastructure, non-physical presence, more workload and the loss of lab courses and the general restriction of social contact have been pointed out as important during the crisis. To the positive characteristics belong the easy participation in class, time savings, home comfort, the possibility to learn, new competences, attending and learning flexibility.

## Materials and Methods

### Study Design

We conducted a longitudinal study in four German universities using an online survey to capture students’ perceptions of technology-mediated teaching throughout the COVID-19 semesters in 2020. Participants in the study were students from selected courses and programs that have been invited to voluntarily take part in the survey. To identify potential *differences between disciplines*, we gathered responses from different subjects being taught. We have used from the beginning defined e-mail distribution lists and the group of potential respondents remained the same throughout the study. Students were asked for their agreement to the respective statements on an administered LimeSurvey. One survey was administered at the beginning of the semester in Germany (April), two surveys during the semester (May and June), and a final survey at the end of the semester (July 2020).

### Measures

The study focused on two main theoretical constructs: (1) (technology) acceptance of e-learning (see Section “Technology Acceptance Model”) and (2) the benefits and disadvantages of e-learning compared with face-to-face or blended learning (see Section “Benefits and Disadvantages of E-Learning”). We relied on pre-tested scales when possible; however, we had to adopt these scales for our study. Furthermore, we collected demographic data and asked open-ended questions to gain deeper insights into students’ perceptions over the semester.

Concerning the first group of acceptance measurements, we used related items from former studies in a comparable context. We adopted the measurement scales for PU, PEOU, PE, ATT, and BI from Lee et al. (2005), as the authors had already pre-tested these constructs for e-learning activities and proven their applicability. As in the original constructs, the items were measured using a 7-point Likert scale. Slight modifications were made to fit items to the investigated e-learning context.

To address the benefits and disadvantages of e-learning, the identified factors (see Section “Benefits and Disadvantages of E-Learning”) were operationalized through a combination of previous studies and the authors’ assessment. As highlighted in the previous chapter, for time flexibility (TF), learning flexibility (LF), and social isolation (SI), the theoretical literature provides several important insights into the factors behind the advantages and disadvantages of technology-mediated teaching environments. Table 2 provides an overview of survey constructs and related measurement items as well as their sources of adoption.

**TABLE 2:** Measurement scale.

**Concept/Context**	**Construct**	**Measurement scale source**
Technology Acceptance	Perceived Usefulness	Adopted from Lee et al. (2005)
	Perceived Ease of Use	Adopted from Lee et al. (2005)
	Perceived Enjoyment	Adopted from Lee et al. (2005)
	Attitude	Developed based on Lee et al. (2005)
	Behavioral Intention	Adopted from Lee et al. (2005)
Benefits and disadvantages e-learning	Learning Flexibility	Modified and further developed from Jefferson and Arnold (2009)
	Time Flexibility	Developed based on Al-Qahtani and Higgins (2013)
	Social Isolation	Modified from Becker et al. (2013)

### Procedure

To identify differences in students’ perceptions over time, we surveyed the same student populations four times during the semester. At University 1, we gathered responses from master’s students in IS, while at Universities 2, 3, and 4, we surveyed participants involved in courses that are part of the music and arts curriculum (bachelor, M&A).

We sent a link to the questionnaire throughout the semester and gathered 875 responses, of which 246 (28%) came from IS students and 629 (72%) from M&A students. We gathered 147 responses in April, 319 in May, 269 in June, and 128 in July. Of the responses, 59% (513) were received from women, 35% (310) came from men, and the remaining 62 (6%) specified another sex or provided no information.

### Data Analysis

Data preparation and analysis were conducted in R with the Stats package, version 3.6.1. Incorrect encodings and values were filtered manually. Throughout the survey, no questions were designated as mandatory. For model testing, only constructs for which all related items were answered were used. Regression model analysis was used to test the individual models. Regression models were estimated using the ordinary least square (OLS) method. The survey constructs were calculated based on the mean values of the respective items. Given the focus of our study, we employed the students’ subject as the control variable in all model constructs (see the section on the differences between student groups). A binary dummy variable indicating the M&A group was used.

Table 3 provides descriptive details for the model constructs. The constructs average values varied. The respondents assessed the ease of using technology-mediated teaching and related technologies as relatively high (avg. 5.2, SD = 1.19) and simultaneously stated that learning with digital technologies did not necessary lead to completely socially isolated work (avg. 3.47, SD = 1.57). The students were almost in agreement regarding the benefits of learning flexibility (avg. 4.92) and time flexibility (avg. 5.01).

**TABLE 3:** Descriptive information on model constructs.

**Construct**	**Min**	**Max**	**Mean**	**SD**	**Mean (SD) – Group IS**	**Mean (SD) – Group M&A**
Perceived usefulness	1	6.67	3.97	1.02	4.64 (0.69)	3.71 (1.01)
Perceived ease of use	1	7	5.2	1.19	5.50 (1.08)	5.09 (1.21)
Behavioral intention	1	7	4.33	1.68	5.35 (1.21)	3.93 (1.68)
Perceived enjoyment	1	7	4.14	1.81	5.35 (1.23)	3.66 (1.78)
Attitude	1	7	4.24	1.56	5.50 (1.04)	3.75 (1.45)
Learning flexibility	1	7	4.92	1.26	5.72 (0.92)	4.60 (1.23)
Time flexibility	1	7	5.01	1.43	5.76 (1.10)	4.72 (1.44)
Social isolation	1	7	3.47	1.57	3.63 (1.11)	3.41 (1.71)

A comparison of the students’ groups revealed that uniformity within the information systems group was larger in almost any of the respective constructs (standard deviation was lower). Moreover, we observed that the agreement was higher for the central model constructs for the group of IS students. Details are discussed in the following sections.

#### Measurement Validity

To ensure the validity of the measurement constructs, two approaches were used. For the new items regarding the benefits and disadvantages of technology-mediated teaching and learning, we first employed an explorative factor analysis (EFA) to assess their suitability to measure related aspects. Apart from the developed items, we assessed the internal validity for all constructs in the model.

Explorative factor analysis has been applied for the constructs related to technology-mediated teaching and learning validity and reliability. A principal component factor analysis with a maximal likelihood estimation rotation was performed on the collected items. The related nine items were employed in a factor analysis, resulting in three constructs. Factor 1 (time flexibility) comprised two items reported on a 7-point Likert scale that explained 30% of the variance with factor loadings from 0.652 to 0.997. Factor 2 (learning flexibility) comprised two items (instead of the three expected, compare Table 4 for the item deleted after the EFA) reported on a 7-point Likert scale that explained 12% of the variance with factor loadings from 0.573 to 0.678. Factor 3 (social isolation) comprised three items reported on a 7-point Likert scale that explained 26% of the variance, with factor loadings from 0.758 to 0.929. Following the results of the EFA, the factors social isolation and time flexibility matched our developed items for each construct. Concerning learning flexibility, the item related to the video lectures (c.f. Table 4) did not match to a significant extent (0.351) and was dropped accordingly.

**TABLE 4:** Assessment of construct reliability.

**Construct**	**CA**	**Measurement instrument/item**
Perceived usefulness	0.69	Teaching with digital learning media will worsen my course grades. Teaching with digital learning media has more advantages than disadvantages. The use of digital learning media in teaching is advantageous overall.
Perceived ease of use	0.62	My lecturers’ instructions on how to use the digital learning media are difficult to follow. It is difficult to learn how to use the digital learning media in the learning process.
Behavioral intention	0.87	I intend to use digital learning media for self-study purposes in the next semester. I intend to use digital learning media in the next semester when preparing projects, papers and assignments.
Perceived enjoyment	0.92	Learning with digital media is pleasant for me. Learning with digital media is fun for me.
Attitude	0.82	I understand the crisis as an opportunity for the spread of digital education in universities. I welcome the increasing relocation of educational processes to virtual space, i.e., presence teaching being replaced by online teaching. I am confident that in the virtual semester, teaching content can be taught without major obstacles.
Learning flexibility	0.88	The use of digital media enables me to learn flexibly. The use of digital media enables me to learn in a self-directed way. *The use of digital media allows me to watch video lectures several times. (Item deleted after EFA)
Time flexibility	0.79	Using digital media saves me time and resources on traveling to university. Using digital media optimizes my time management.
Social isolation	0.89	The use of digital media leads to socially isolated work. The use of digital media changes my direct communication with fellow students. The use of digital media changes my direct communication with lecturers.

Lastly, the internal validity was assessed for all constructs. The established group of technology acceptance constructs was only tested for their internal validity through Cronbach’s alphas. Table 4 provides an overview of the survey constructs internal validity and the survey items used. Apart from the PU and PEOU constructs, internal validity was fulfilled for the constructs employed (≥0.7).

## Results

**FIGURE 2:**
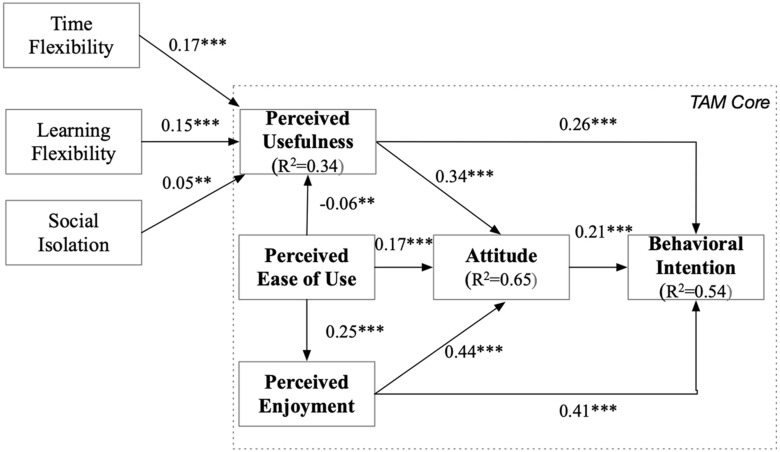
TAM test results, including LF, TF, and SI as influencing variables. ** Significant at the 0.01-level and *** significant at the 0.001-level.

The overall results of the structural model test are shown in Figure 2. The model accounts for 65% of the variance in ATT and 54% of the variance in BI.

For all the model constructs, the significant factors were identified with the survey data. Table 5 provides an overview of the hypotheses and the related results. With the exception of H1 (PEOU –> PU), all TAM hypotheses could be verified in our sample.

**TABLE 5:** Summary of hypothesis tests.

**Hypothesis**	**Coefficient**	**Test**
	**(*p*-value)**	**result**
H1: Perceived Ease of Use -> Perceived Usefulness	−0.06**	Not confirmed
H2: Perceived Ease of Use -> Attitude	0.17***	Confirmed
H3: Perceived Usefulness -> Attitude	0.34***	Confirmed
H4: Perceived Enjoyment -> Attitude	0.44***	Confirmed
H5: Perceived Ease of Use -> Perceived Enjoyment	0.25***	Confirmed
H6: Attitude -> Behavioral Intention	0.21***	Confirmed
H7: Perceived Enjoyment -> Behavioral Intention	0.41***	Confirmed
H8: Perceived Usefulness -> Behavioral Intention	0.74***	Confirmed

** Significant at the 0.01-level and *** significant at the 0.001-level.

### Effects of Benefits and Disadvantages of E-Learning on PU

**FIGURE 3:**
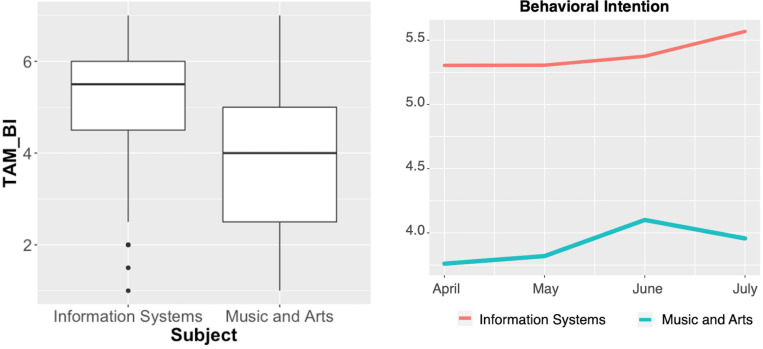
Behavioral intention between subject groups and over time.

Two of the items in PU directly address the perception of the benefits or advantages of technology-mediated teaching. The third deals with the direct output of learning, which is related to its perceived effectiveness (cf. Table 4). Thus, we analyzed the data in view of the potential relations between the perceived benefits and disadvantages of technology-mediated teaching and PU. Based on our empirical results, we were interested in identifying the sentiments underlying students’ perceptions of the usefulness of technology-mediated teaching. We therefore extended the TAM core model with the three new factors influencing PU, as presented in Figure 3.

Furthermore, we conducted a regression analysis of PU over time, as illustrated in Table 6. The effects of TF, LF, and SI explained 34% of the variance in PU in the model test (Figure 3), as well as up to 35% of the variance in PU over time (Table 6), with a very low explanation rate in May, which was also the only month when SI had a significant effect on PU.

**TABLE 6:** TAM regression explaining perceived usefulness.

**Coefficients**	***R*^2^**	**Time flexibility**	**Learning flexibility**	**Social isolation**
April	0.29	0.25***	0.12.	0.09
May	0.17	0.21***	0.07	0.09*
June	0.35	0.17**	0.34***	0.01
July	0.35	0.24**	0.20*	0.06

* Significant at the 0.05-level, ** significant at the 0.01-level, and *** significant at the 0.001-level.

### Differences in the Perceptions of IS and M&A Students

To identify the differences between the two student groups, a Kruskal–Wallis test was performed. As a non-parametric test, the approach allowed us to identify differences among our subsamples of different sizes. Table 7 depicts the test results for the model constructs.

**TABLE 7:** Identified differences in the perception between the students.

**Construct**	***p*-value Kruskal–Wallis test**	**Differences between M&A and IS**
PU	<2.2e^–16^***	Identified
PEU	<8.54^–6^***	Identified
PE	<2.2e^–16^***	Identified
ATT	<2.2e^–16^***	Identified
BI	<2.2e^–16^***	Identified
SI	<0.0003***	Identified
TF	<2.2e^–16^***	Identified
LF	<2.2e^–16^***	Identified

*** Significant at the 0.001-level.

Overall, all central model constructs vary between the student’s subject. Moreover, in general compared with IS students, M&A students have more negative perceptions of almost all model constructs.

### Differences in Acceptance Over Time

The differences in the central model constructs were analyzed in terms of variations over time and between subject groups. Figure 3 shows the results for BI over time and between subject groups as generally higher for IS students and indicates further differences over time. For IS students, the analysis results reveal increased BI over time toward the end of the semester. For the M&A group, we found similar increased BI over time; a slight decline was identified at the end of the semester.

**FIGURE 4:**
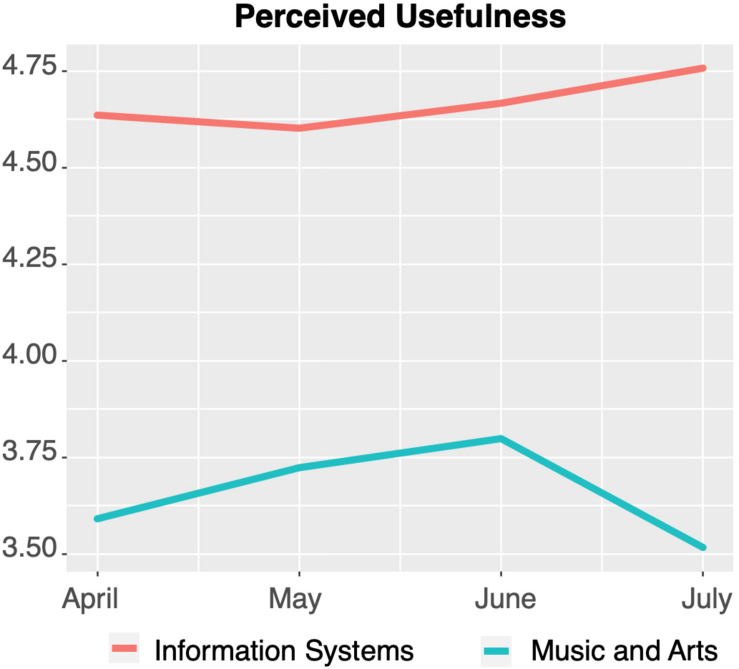
Perceived usefulness of technology-mediated teaching over time.

**FIGURE 5:**
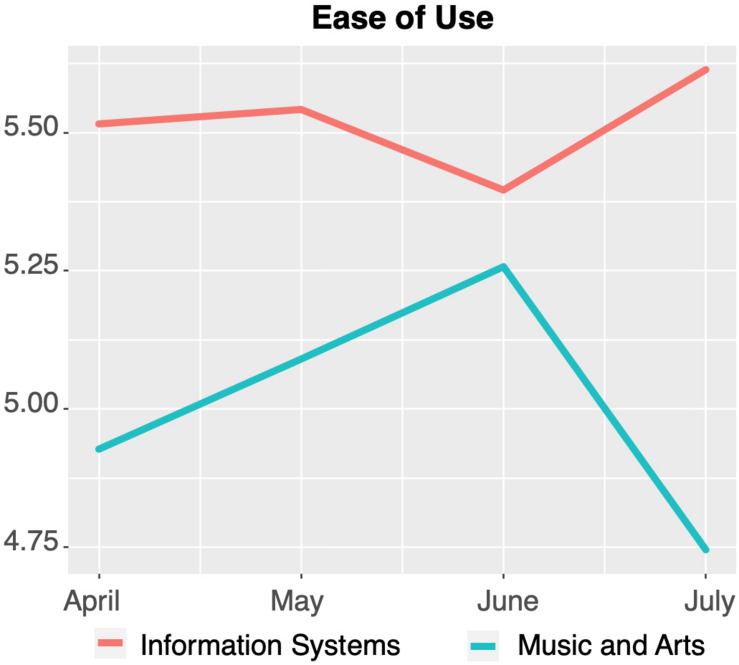
Perceived ease of use of technology-mediated teaching over time.

**FIGURE 6:**
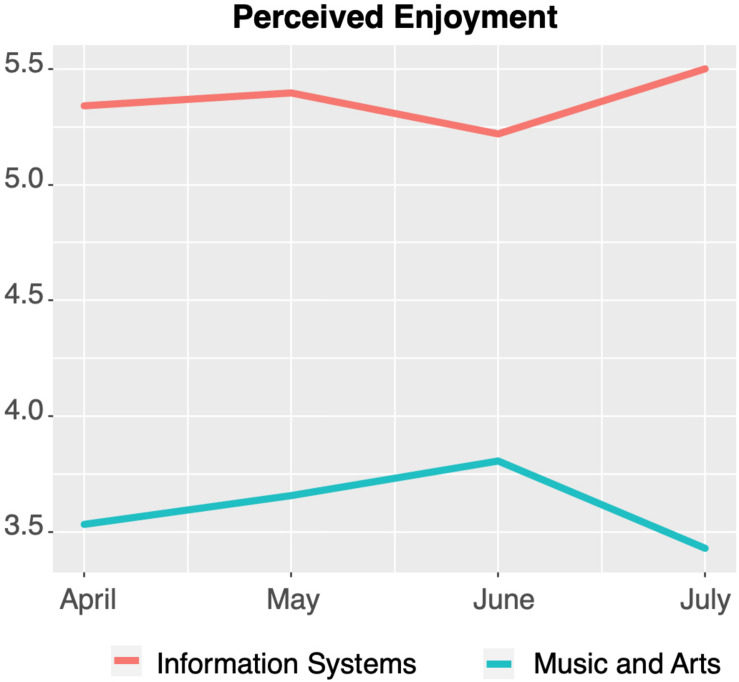
Perceived enjoyment of technology-mediated teaching over time.

The same tendency in development over time and in significant (see Table 7) differences between the subject groups was observed with regard to PU, PEOU, and PE (visualized in Figures 4–6, respectively).

As shown in Table 8, the model explains up to 59% of the variance in BI for IS students and up to 52% for M&A students. The effect of PU was not significant for both student groups at the beginning and at the end of semester and remained constantly non-significant for IS students. For M&A students, PU was very significant in the middle of the semester. The effect of ATT was significant in the beginning and the middle of the semester (June) for IS students but weakened over time. For M&A students, the effect of ATT also varied during the semester, being significant in the second month and at the end of the semester. The strongest and most constant significant effect was found for PE in both groups.

**TABLE 8:** TAM regression results explaining BI.

	**Information systems**		**Music and Arts**	
	***R*^2^**	**Coefficient**	***R*^2^**	**Coefficient**
April	0.56		0.52	
TAM_PU		–0.14		0.40
TAM_Attitude		0.34***		–0.11
TAM_PE		0.57***		0.65***
May	0.52		0.40	
TAM_PU		0.02		0.29**
TAM_Attitude		0.23		0.21**
TAM_PE		0.59***		0.37***
June	0.59		0.48	
TAM_PU		0.58*		0.40***
TAM_Attitude		0.40*		0.12
TAM_PE		0.24		0.38***
July	0.49		0.52	
TAM_PU		–0.12		0.27
TAM_Attitude		0.11		0.43***
TAM_PE		0.75**		0.32**

* Significant at the 0.05-level, ** significant at the 0.01-level, and *** significant at the 0.001-level.

## Discussion of the Results

In this study, we identified differences in the perceptions of the investigated subject groups and over time.

The first research question (RQ1) could be answered positively: For all constructs of our model, the results show significant differences toward completely technology-mediated teaching depending on the discipline of study. In general, for all constructs, M&A students answer more negatively than IS, which leads to the conclusion that they will not accept (complete) technology-mediated teaching to the same extent as IS students. This supports, *inter alia*, our theoretical findings, especially the findings of Pike et al. (2012), which emphasized that students’ academic majors were significantly related to levels of engagement, which is influenced by their acceptance and learning outcomes. IS students in our study furthermore enjoyed the technology-mediated teaching more, although social isolation was the most negatively indicated by both groups. Neumann (2001) emphasizes a strong influence of disciplines on students’ learning and behavior, and Nelson et al. (2011) point out differences in facets of digital literacy and competency. Our findings empirically support disciplinary differences in acceptance of technical-mediated teaching between M&A and IS students. We assume a higher acceptance of IS to be a result of the appropriateness of the medium for the subjects’ content and the confidence that the content of their lecture can be conveyed technologically, as well as based on general openness toward technology-mediated teaching. The higher acceptance of virtual classroom format might also be a result of the general tendency for people to adopt familiar formats more easily (cf. Janssen et al., 2009); that is, in the present case, IS students were more familiar with technologies and virtual environments than M&A students, and, thus, possibly influencing the corresponding acceptance.

To answer the second research question (RQ2), we analyzed differences over time and between the two groups. The results show that this research question was also positively answered: The students’ attitudes toward completely technology-mediated teaching changed over time during the COVID-19 semester. Especially in the last month of the semester, a decline in all constructs was apparent for the M&A group. One reason for this finding may be that at this time, the loosening of social isolation had begun, and face-to-face teaching was possible again, clearly demonstrating its advantages for this group compared with completely technology-mediated teaching. A reason for M&A students’ perception of technology-mediated teaching as much less suitable for conveying their learning content could be the lack of opportunities for laboratories and studios, audience response (in music and theater), and practical work in technology-mediated teaching, which are a main focus of their curriculum. We assume that the type of knowledge imparted in the curriculum is also responsible for these differences.

Further, in the context of RQ2, we measured the effect of PE, ATT, and PU on BI separately for both groups using a regression analysis. Even if both groups did not rate the PU as high, the usefulness of technology-mediated teaching did not significantly affect the intentional behavior for any of the months of the survey. PU seemed to be important only for M&A students in the middle of the semester. This could be explained by the fact that at this time, M&A students had gained enough experience and recognized that the contents of their study cannot be transferred properly enough in a technology-mediated teaching environment. The intrinsic factor – enjoyment – however, has a decisive importance, with the remaining strong influencing effect for both groups. Further, the results show that PE was much lower for M&A students. This should be an object of further investigation focusing on variables influencing enjoyment of technology-mediated teaching. To address the special situation during this empirical study, the attitude toward technology-mediated teaching was placed in a close relation to the COVID-19 crisis. The effects on BI over time should therefore also be discussed in the context of the crisis. In the middle of the semester, the BI of both groups was significantly affected by the perception of the students regarding the influence of the crisis on the future digitalization of learning processes as well as on the current semester. At the end of the semester, however, IS and M&A students’ responses developed in different directions – the experiences of IS students reduced this significant effect, although for the second group, the effect remained significant at the end. For this group, the lessons learned during the crisis are also more negative.

Besides the results related to RQ1 and RQ2, we provided results with regard to the TAM, which are in alignment with the results of our main theoretical underlying basis, Lee et al. (2005) as well as Venkatesh and Davis (2000). Significant effects could be confirmed in TAM core constructs. We identified in contrast to prior research an inverse effect of PEOU on PU. This might be explained by the fact that this was conducted during the COVID-19 situation, not voluntary, and we did not question the use of a specific technology, but rather technology-mediated teaching in general.

We identified a change of acceptance over time. Shehzadi et al. (2020) investigated the influence of e-learning of Pakistani public and private university students on their satisfaction in the context of the pandemic. Therein, a positive dependence of students’ satisfaction with information and communication technologies, e-service quality, and e-information quality as influencing factors of students’ e-learning experience was identified. This implies that specific technologies, the service quality, which is, for example, technological smoothness and a high degree of usability, as well as teacher and teaching characteristics, might also be decisive factors for consideration when (re)designing technology-mediated teaching and learning and, thus, addressing student acceptance.

We extended prior research introducing the new variables LF, TF, and SI to TAM and showed how they influence the PU of technology-mediated teaching learning. The possibilities to learn from home, save travel time, and access (video-recorded) lectures independently of time and place are universal benefits of technology-mediated teaching and learning that have gained importance under the special conditions of the lockdown period. Thus, it is not surprising that LF and TF were positively related to PU. The effect of LF was identified as significant in June (Table 6). TF had a strong effect during the whole lockdown period. Regarding the perceived disadvantages of technology-mediated teaching, our results show that SI had a surprisingly positive effect on PU. This could be explained by the situation of the complete lockdown, without alternatives to learning and direct exchange. There is evidence that the willingness to perceive technology-mediated teaching and learning as equivalent to face-to-face teaching and learning is greatest when offered without alternatives (Mehra and Omidian, 2011). The impact of SI on PU was strongest in the second month of the lockdown. This may be the result of the overall phase characteristics: during the second time, it became clear that the crisis would last longer, but the frustration about the social isolation was not yet too great by comparison.

## Implications for Teaching Practice, Educational Technology and Further Research

The results of this study on technology acceptance during the virtual COVID-19 semester in Germany are important in both the short and the long term. We point out three areas of implications: teaching practice and organization, educational technology, and research.

### Teaching Practice and Organization

Our study was conducted in a situation of immediate switch from physical presence to technology-mediated teaching. The extreme circumstances were a big challenge; however, they provided important evidence about technology-mediated teaching at universities. In the current course of the pandemic, the fall-winter 20/21 semester is equally or at least partially technology-mediated. In this respect, the findings can help improve teaching directly, especially regarding the differences in the perceptions of the subjects of study.

The differences between the student groups need to be taken into consideration by the teachers when designing virtual teaching and learning environments and conducting teaching. For example, different formats, such as breakout sessions in smaller groups, could be used. Furthermore, specific sensitization to the advantages or necessity of the formats can be applied or the degree of interactivity within the sessions adjusted. To this end, teachers should develop competencies, not only regarding the use of technical tools but also new didactic and methodological skills. Further, the overlap of technological, pedagogical and content knowledge leads to new kinds of interrelated knowledge (Mishra and Koehler, 2006; Archambault and Crippen, 2009; Schmid et al., 2020), which are gaining importance in the context of teachers’ education and professional development. The transfer of knowledge through teaching must not occur in such a way that a single technique implies innovation. It is much more challenging for lecturers to demonstrate their methodological and professional competencies through the use of media in the same way as in face-to-face teaching. The initial experiences during the COVID-19 lockdown have shown both possibilities and limitations. The students’ direct feedback is all the more important to better exploit the potential of technology-mediated teaching in the future.

In the long term, not only direct teaching practices but also the organization of the teaching processes at the universities as a whole should be taken into consideration. Customized approaches, which differ in respective share of online and offline teaching and learning formats, should be considered for students of different subjects. Whereas, for example, IS students are more familiar with virtual environments, it is assumed that they are more likely to accept and manage the switch to fully virtual learning formats. By contrast, M&A students who are generally assumed to be less familiar with virtual environments may show less acceptance of related formats. Moreover, the appropriateness of virtual teaching and learning may also generally vary among subjects. The acceptance of virtual learning formats should not be considered as similar for all students simply referring to their age/generation. We argue for a consideration of their familiarity and competences with related technologies as well as their technological affinity, which varies among subjects. Moreover, we surmise that personal interaction may not be fully substituted through virtual formats. Hybrid teaching forms seem to become most promising for the future of learning and teaching at universities (Vladova et al., 2020). Therefore, administrative and organizational changes and reorganization of (well established) practices become necessary. These will involve adjustment and further development of the curriculum, stable and trustful technological infrastructure, organization of learning results assessment, as well as the development of a new culture of technology-mediated teaching, including netiquette, behavioral norms, and standards.

### Educational Technology

The differences between the student groups clearly show that the use of technologies and the design of technology-mediated teaching offerings should address the specific needs of different study subjects. At the time of the study, communication platforms such as Zoom, Cisco Webex, or Big Blue Button were mainly used for teaching, as well as Moodle as a learning platform for organization of the teaching process. Against this background, the direct user feedback in our study includes important hints for educational technology (EdTech) companies. Currently, these companies mostly focus on the development of learning courses for individual use, pointing out the role of artificial intelligence (AI) and learning analytics. However, the results show the immense importance of the differences in the field of study during the transfer of knowledge in an academic environment. This can be addressed by short- and long-term solutions and may lead to innovative concepts and products, whereby the role of the teacher remains central for the transfer of specific study content. However, students can acquire different content in a completely self-directed and self-organized way. The curriculum of the two groups in our study can be used, among other things, to identify the subject-specific needs of the students.

### Research

In the long term, the effects of LF, TF, and SI should be empirically tested and investigated by further research in a COVID-19 neutral situation. Furthermore, the changes in the TAM constructs over time refer to the influence of experience within the acceptance model in education. Thus, future research should investigate whether this experience can influence students’ habits and, through this, their acceptance of face-to-face teaching. This is relevant for the phase of returning to direct face-to-face teaching after the crisis, but much more in the long term as university teaching becomes increasingly technology-mediated.

We also identified implications for further research in the context of knowledge management. The results of the study indicate a relationship between the nature of knowledge transferred during the teaching process and the acceptance of technology-mediated teaching. When the shift to the technology-mediated learning environment is considered, the nature of knowledge and how it is transferred comes to the forefront (Vladova et al., 2020). The knowledge management literature points out the critical distinction between tacit knowledge (person-bound) and explicit knowledge (not person-bound) (Polanyi, 1966). Whereas explicit knowledge can be transferred in the context of communication processes with the help of numbers, pictures, or language, tacit knowledge is personal and context-specific (Nonaka and Takeuchi, 1995). Therefore, tacit knowledge is difficult to communicate (Nonaka and Takeuchi, 1995) and can be transferred only partly and by common application and practice. For example, Polanyi (1958, p. 92) posits: “Although the expert (…) can indicate their clues and formulate their maxims, they know many more things than they can tell, knowing them only in practice, as instrumental particulars, and not explicitly, as objects.”

Next, during our data analysis, we found some implications for research on the topic of innovation diffusion (Rogers, 2010), as IS students can probably be described as early adaptors and M&A students as the late majority. IS-students can thus be used as a test audience as well as ambassadors for a new learning technology solution. Thus, they would have a trendsetting role within universities. Thanks to their high acceptance, new technologies can be tried out without fear of resistance and their advantages can be recognized.

We also believe that our study could be of interest in the interdisciplinary research field, especially in the context of digital-mediated team, net, and project work. At this point, the experiences and needs of M&A students are especially important to explore. Experiments as well as surveys on these types of teamwork in the university context can provide necessary information on how technology-mediated teaching should be appropriately designed for this user group. This necessitates scientific collaboration between work psychologists, computer scientists and educators.

## Conclusion

Although a study of this scale cannot be wholly representative of the entire higher education sector, it has provided views from two different disciplines, that is, M&A as well as IS, on the acceptance of technology-mediated teaching and learning in four universities in Germany. Motivated by the need to understand the underlying drivers of student adoption of digital-mediated learning during the COVID-19 semester, we applied the TAM in a longitudinal study and incorporated three new variables (LF, TF, and SI) influencing PU into the TAM. Furthermore, we identified differences between the subject groups regarding their perceived acceptance of digital-mediated teaching and showed the changes in BI over time for both student groups. We used a validated construct for acceptance. However, as we were aware of the specifics of the situation – social isolation and no alternative to the use of technology – we first tested the hypotheses using our sample.

Our study also has some limitations. First, it was conducted under the special circumstances of complete social isolation in every area of life, which has an influence on the results. Furthermore, we summarized the M&A group in the evaluation without consideration for the differences within it (e.g., music, theater, architecture, visual communication). Given the urgency and the circumstances of the situation of our empirical research context, we furthermore did not have the opportunity to directly examine the organizational situation at the participating universities. However, we included questions about digital platforms and tolls, as well as open-ended questions about students’ perceptions of the performance of their teachers. Thus, we addressed organizational and technical issues and their impact on student acceptance. The answers to these questions are not the focus of this paper; however, they will help us to place the model in connection with the specific framework conditions at the universities and to analyze the answers more in depth.

In following up on our data analysis, our future research will especially address the changes on the individual level over time, further data collection in the current semester (fall-winter 20/21), and the analysis of the gathered qualitative data of the answers to the open-ended questions. These efforts will allow us to gain further information on students’ perceptions of technology-mediated teaching during the semester.

## Data Availability Statement

The raw data supporting the conclusions of this article will be made available by the authors, without undue reservation.

## Ethics Statement

Ethical approval was not provided for this study on human participants because this was an anonymous survey, we used the own server for data administration. The participants provided their informed consent to participate in this study.

## Author Contributions

GV developed the idea for this empirical research and was involved in all steps of the study and the manuscript preparation. GV and AU prepared the instrument applied in the empirical study. Both prepared a large part of the introduction, theory, and discussion of the results. GV was mainly responsible for the implications, AU for the conclusion. BB carried out the data analysis and described its procedure and results. NG was actively involved in the implementation of the survey and was responsible for the internal review process. All authors contributed to the article and approved the submitted version.

## Conflict of Interest

The authors declare that the research was conducted in the absence of any commercial or financial relationships that could be construed as a potential conflict of interest.

## Attachment 1

H1: There is a positive relationship between perceived ease of use and perceived usefulness.
H2: There is a positive relationship between perceived ease of use and attitude.
H3: There is a positive relationship between perceived usefulness and attitude.
H4: There is a positive relationship between perceived enjoyment and attitude.
H5: There is a positive relationship between perceived ease of use and perceived enjoyment.
H6: There is a positive relationship between attitude and behavioral intention.
H7: There is a positive relationship between perceived enjoyment and behavioral intention.
H8: There is a positive relationship between perceived usefulness and behavioral intention.

## References

[B1] Al-alak B. A. Alnawas I. A. M. (2011). Measuring the acceptance and adoption of E-Learning by academic staff. *Knowl. Manage. E-Learn. Int. J.* 3 201–221. 10.34105/j.kmel.2011.03.016

[B2] Al-Azawei A. Parslow P. Lundqvist K. (2017). Investigating the effect of learning styles in a blended e-learning system: an extension of the technology acceptance model (TAM). *Austr. J. Educ. Technol.* 33:2 10.14742/ajet.2741

[B3] Al-Harbi K. A.-S. (2011). e-Learning in the Saudi tertiary education: potential and challenges. *Appl. Comput. Inform.* 9 31–46. 10.1016/j.aci.2010.03.002

[B4] Al-Qahtani A. A. Higgins S. E. (2013). Effects of traditional, blended and e-learning on students’ achievement in higher education. *J. Comput. Assist. Learn.* 29 220–234. 10.1111/j.1365-2729.2012.00490.x

[B5] Archambault L. Crippen K. (2009). Examining TPACK among K-12 online distance educators in the United States. *Contemp. Issues Technol. Teacher Educ.* 9 71–88.

[B6] Becker K. Newton C. Sawang S. (2013). A learner perspective on barriers to e-learning. *Austr. J. Adult Learn.* 53:211.

[B7] Berge Z. L. Giles L. (2006). Implementing and sustaining e-learning in the workplace. *Int. J. Inform. Commun. Technol. Educ. (IJICTE)* 2 64–75. 10.4018/jicte.2006100106

[B8] Berk R. A. (2009). Multimedia teaching with video clips: TV, movies, Youtube, and mtvU in the college classroom. *J. Technol. Teach. Learn.* 5 1–21.

[B9] Biglan A. (1973). The characteristics of subject matter in different academic areas. *J. Appl. Psychol.* 57 195–203. 10.1037/h0034701

[B10] Brown L. Murphy E. Wade V. (2006). Corporate eLearning: human resource development implications for large and small organizations. *Hum. Resour. Dev. Int.* 9 415–427. 10.1080/13678860600893607

[B11] Cameron L. (2017). How learning designs, teaching methods and activities differ by discipline in Australian universities. *J. Learn. Design* 10 69–84. 10.5204/jld.v10i2.289

[B12] Camilleri M. A. (2020). “Strategic dialogic communication through digital media during COVID-19 crisis,” in *Strategic Corporate Communication in the Digital Age*, ed. Camilleri M. A. (Bingley: Emerald).

[B13] Carlson S. (2005). The net generation in the classroom. *Chronicle Higher Educ.* 52 34–37.

[B14] Cashin W. E. Downey R. G. (1995). “Disciplinary differences in what is taught and in students’ perceptions of what they learn and of how they are taught,” in *Disciplinary Differences in Teaching and Learning: Implications for Practice*, eds Hativa N. Marincovich M. (San Francisco, CA: Jossey-Bass), 81–92. 10.1002/tl.37219956412

[B15] Crawford J. Butler-Henderson K. Rudolph J. Malkawi B. Glowatz M. Burton R. (2020). COVID-19: 20 countries’ higher education intra-period digital pedagogy responses. *J. Appl. Learn. Teach.* 3 1–20. 10.1080/1475939x.2020.1866654

[B16] Davis F. D. Bagozzi R. P. Warshaw P. R. (1989). User acceptance of computer technology: a comparison of two theoretical models. *Manage. Sci.* 35 982–1003. 10.1287/mnsc.35.8.982 19642375

[B17] Davis R. Wong D. (2007). Conceptualizing and measuring the optimal experience of the eLearning environment^∗^. *Decis. Sci. J. Innovat. Educ.* 5 97–126. 10.1111/j.1540-4609.2007.00129.x

[B18] Derouin R. E. Fritzsche B. A. Salas E. (2005). E-learning in organizations. *J. Manage.* 31 920–940. 10.1177/0149206305279815

[B19] Gimson A. Bell J. (2007). E-learning: your flexible development friend? *Dev. Learn. Organ. Int. J.* 21 7–9. 10.1108/14777280710828558

[B20] Hameed S. Badii A. Cullen A. J. (2008). “Effective e-learning integration with traditional learning in a blended learning environment,” in *Proceedings of the European and Mediterranean Conference on Information Systems*, (Dubai), 25–26.

[B21] Hill N. S. Wouters K. (2010). Comparing apples and oranges: toward a typology for assessing e-learning effectiveness. *Res. Pers. Hum. Resour. Manage.* 29:201. 10.1108/s0742-7301(2010)0000029008 21381016

[B22] Howe N. (2000). *Millennials Rising: The Next Great Generation.* New York, NY: Vintage.

[B23] Hu P. J. Chau P. Y. Sheng O. R. L. Tam K. Y. (1999). Examining the technology acceptance model using physician acceptance of telemedicine technology. *J. Manage. Inform. Syst.* 16 91–112. 10.1080/07421222.1999.11518247

[B24] Janssen J. Erkens G. Kirschner P. A. Kanselaar G. (2009). Influence of group member familiarity on online collaborative learning. *Comput. Hum. Behav.* 25 161–170. 10.1016/j.chb.2008.08.010

[B25] Jefferson R. N. Arnold L. W. (2009). Effects of virtual education on academic culture: perceived advantages and disadvantages. *Online Subm.* 6 61–66.

[B26] Jones C. Ramanau R. Cross S. Healing G. (2010). Net generation or digital natives: is there a distinct new generation entering university? *Comput. Educ.* 54 722–732. 10.1016/j.compedu.2009.09.022

[B27] Kamarianos I. Adamopoulou A. Lambropoulos H. Stamelos G. (2020). Towards an understanding of university students’ response in times of pandemic crisis (COVID-19). *Eur. J. Educ. Stud.* 7:7 10.46827/ejes.v7i7.3149

[B28] Karalis T. Raikou N. (2020). Teaching at the times of COVID-19: inferences and implications for higher education pedagogy. *Int. J. Acad. Res. Bus. Soc. Sci.* 10:5 10.6007/IJARBSS/v10-i5/7219

[B29] Lee M. K. Cheung C. M. Chen Z. (2005). Acceptance of internet-based learning medium: the role of extrinsic and intrinsic motivation. *Inform. Manage.* 42 1095–1104. 10.1016/j.im.2003.10.007

[B30] Liu S.-H. Liao H.-L. Peng C.-J. (2005). Applying the technology acceptance model and flow theory to online e-learning users’ acceptance behaviour. *Issues Inform. Syst.* 2:7.

[B31] Mehra V. Omidian F. (2011). Examining students’ attitudes towards E-learning: a case from India (No. 2). *Malays. J. Educ. Technol.* 11:6.

[B32] Mishra P. Koehler M. J. (2006). Technological pedagogical content knowledge: a framework for teacher knowledge. *Teachers College Rec.* 108 1017–1054. 10.1111/j.1467-9620.2006.00684.x

[B33] Mohammadi H. (2015). Investigating users’ perspectives on e-learning: an integration of TAM and IS success model. *Comput. Hum. Behav.* 45 359–374. 10.1016/j.chb.2014.07.044

[B34] Nelson K. Courier M. Joseph G. W. (2011). An investigation of digital literacy needs of students. *J. Inform. Syst. Educ.* 22 95–110.

[B35] Neumann R. (2001). Disciplinary differences and university teaching. *Stud. High. Educ.* 26 135–146. 10.1080/03075070120052071

[B36] Neumann R. Parry S. Becher T. (2002). Teaching and learning in their disciplinary contexts: a conceptual analysis. *Stud. High. Educ.* 27 405–417. 10.1080/0307507022000011525

[B37] Nonaka I. Takeuchi H. (1995). *The Knowledge Creating Company – How Japanese Companies Create the Dynamic Innovation.* Oxford: Oxford University Press.

[B38] Nuere S. de Miguel L. (2020). The digital/technological connection with COVID-19: an unprecedented challenge in University teaching. *Technol. Knowl. Learn.* 10.1007/s10758-020-09454-6 [Epub ahead of print].

[B39] Owusu-Fordjour C. Koomson C. K. Hanson D. (2020). The impact of Covid-19 on learning-the perspective of the Ghanaian student. *Eur. J. Educ. Stud.* 7 88–101.

[B40] Park S. Y. (2009). An analysis of the technology acceptance model in understanding University students’ behavioral intention to use e-Learning. *J. Educ. Technol. Society* 12 150–162.

[B41] Pike G. R. Smart J. C. Ethington C. A. (2012). The mediating effects of student engagement on the relationships between academic disciplines and learning outcomes: an extension of Holland’s theory. *Res. High. Educ.* 53 550–575. 10.1007/s11162-011-9239-y

[B42] Pituch K. A. Lee Y. (2006). The influence of system characteristics on e-learning use. *Comput. Educ.* 47 222–244. 10.1016/j.compedu.2004.10.007

[B43] Polanyi M. (1958). *Personal Knowledge: Towards a Post-Critical Philosophy*, Vol. 1 London: Routledge.

[B44] Polanyi M. (1966). *The Tacit Dimension.* NewYork, NY: Doubleday.

[B45] Pynoo B. Devolder P. Tondeur J. van Braak J. Duyck W. Duyck P. (2011). Predicting secondary school teachers’ acceptance and use of a digital learning environment: a cross-sectional study. *Comput. Hum. Behav.* 27 568–575. 10.1016/j.chb.2010.10.005

[B46] Radanliev P. De Roure D. Walton R. (2020a). Data mining and analysis of scientific research data records on Covid-19 mortality, immunity, and vaccine development-In the first wave of the Covid-19 pandemic. *Diabetes Metab. Syndr. Clin. Res. Rev.* 14 1121–1132. 10.1016/j.dsx.2020.06.063 32659695 PMC7335244

[B47] Radanliev P. De Roure D. Walton R. Van Kleek M. Montalvo R. M. Santos O. (2020b). COVID-19 what have we learned? The rise of social machines and connected devices in pandemic management following the concepts of predictive, preventive and personalized medicine. *EPMA J.* 11 311–332. 10.1007/s13167-020-00218-x 32839666 PMC7391030

[B48] Ramaley J. A. Zia L. (2005). “The real versus the possible: closing the gaps in engagement and learning,” in *Educating the Net Generation*, eds Oblinger D. Oblinger J. (Boulder, CO: EDUCAUSE).

[B49] Rogers E. M. (2010). *Diffusion of Innovations.* NewYork, NY: Simon and Schuster.

[B50] Schmid M. Brianza E. Petko D. (2020). Developing a short assessment instrument for technological pedagogical content knowledge (TPACK. xs) and comparing the factor structure of an integrative and a transformative model. *Comput. Educ.* 157:103967 10.1016/j.compedu.2020.103967

[B51] Selim H. M. (2003). An empirical investigation of student acceptance of course websites. *Comput. Educ.* 40 343–360. 10.1016/S0360-1315(02)00142-2

[B52] Shah S. Diwan S. Kohan L. Rosenblum D. Gharibo Ch Soin A. (2020). The technological impact of COVID-19 on the future of education and health care delivery. *Pain Physician* 23 S367–S380. 32942794

[B53] Shehzadi S. Nisar Q. A. Hussain M. S. Basheer M. F. Hameed W. U. Chaudhry N. I. (2020). The role of digital learning toward students’ satisfaction and university brand image at educational institutes of Pakistan: a post-effect of COVID-19. *Asian Educ. Dev. Stud.* 10.1108/AEDS-04-2020-0063 [Epub ahead-of-print].

[B54] Šumak B. Heričko M. Pušnik M. (2011). A meta-analysis of e-learning technology acceptance: the role of user types and e-learning technology types. *Comput. Hum. Behav.* 27 2067–2077. 10.1016/j.chb.2011.08.005

[B55] Tarhini A. Hone K. Liu X. (2013). Extending the TAM model to empirically investigate the students’ behavioural intention to use e-learning in developing countries. *Sci. Inform. Conf.* 2013 732–737.

[B56] Venkatesh V. Davis F. D. (2000). A theoretical extension of the technology acceptance model: four longitudinal field studies. *Manage. Sci.* 46 186–204. 10.1287/mnsc.46.2.186.11926 19642375

[B57] Venkatesh V. Morris M. G. Davis G. B. Davis F. D. (2003). User acceptance of information technology: toward a unified view. *MIS Q.* 27 425–478. 10.2307/30036540

[B58] Venkatesh V. Thong J. Y. Xu X. (2012). Consumer acceptance and use of information technology: extending the unified theory of acceptance and use of technology. *MIS Q.* 36 157–178. 10.2307/41410412

[B59] Vladova G. Ullrich A. Bender B. Gronau N. (2020). “A critical review of the COVID-19 semester,” in *Proceedings of the TECH-EDU Conference Series, December 2020, online.*

[B60] Vo M. H. Zhu C. Diep A. N. (2020). Students’ performance in blended learning: disciplinary difference and instructional design factors. *J. Comput. Educ.* 7 487–510. 10.1007/s40692-020-00164-7

[B61] Watermeyer R. Crick T. Knight C. Goodall J. (2020). COVID-19 and digital disruption in UK universities: afflictions and affordances of emergency online migration. *High. Educ.* 10.1007/s10734-020-00561-y [Epub ahead of print]. 32836334 PMC7269686

[B62] Welsh E. T. Wanberg C. R. Brown K. G. Simmering M. J. (2003). E-learning: emerging uses, empirical results and future directions. *Int. J. Train. Dev.* 7 245–258. 10.1046/j.1360-3736.2003.00184.x

[B63] Xu D. Jaggars S. S. (2014). Performance gaps between online and face-to-face courses: differences across types of students and academic subject areas. *J. High. Educ.* 85 633–659. 10.1080/00221546.2014.11777343

[B64] Zheng J. Li S. Zheng Y. (2017). Students’ technology acceptance, motivation and self-efficacy towards the eSchoolbag: an exploratory study. *Int. J. Infonomics* 10 1350–1358. 10.20533/iji.1742.4712.2017.0165

